# miR-206 Represses Hypertrophy of Myogenic Cells but Not Muscle Fibers via Inhibition of HDAC4

**DOI:** 10.1371/journal.pone.0073589

**Published:** 2013-09-02

**Authors:** Catherine E. Winbanks, Claudia Beyer, Adam Hagg, Hongwei Qian, Patricio V. Sepulveda, Paul Gregorevic

**Affiliations:** 1 Division of Metabolism and Obesity, Baker IDI Heart and Diabetes Institute, Melbourne, Australia; 2 Centre for Physical Activity and Nutrition Research, School of Exercise and Nutrition Sciences, Deakin University, Burwood, Australia; 3 Department of Neurology, The University of Washington School of Medicine, Seattle, Washington, United States of America; 4 Department of Biochemistry and Molecular Biology, Monash University, Melbourne, Australia; 5 Department of Physiology, the University of Melbourne, Parkville, Australia; University of Minnesota, United States of America

## Abstract

microRNAs regulate the development of myogenic progenitors, and the formation of skeletal muscle fibers. However, the role miRNAs play in controlling the growth and adaptation of post-mitotic musculature is less clear. Here, we show that inhibition of the established pro-myogenic regulator miR-206 can promote hypertrophy and increased protein synthesis in post-mitotic cells of the myogenic lineage. We have previously demonstrated that histone deacetylase 4 (HDAC4) is a target of miR-206 in the regulation of myogenic differentiation. We confirmed that inhibition of miR-206 de-repressed HDAC4 accumulation in cultured myotubes. Importantly, inhibition of HDAC4 activity by valproic acid or sodium butyrate prevented hypertrophy of myogenic cells otherwise induced by inhibition of miR-206. To test the significance of miRNA-206 as a regulator of skeletal muscle mass *in vivo*, we designed recombinant adeno-associated viral vectors (rAAV6 vectors) expressing miR-206, or a miR-206 “sponge,” featuring repeats of a validated miR-206 target sequence. We observed that over-expression or inhibition of miR-206 in the muscles of mice decreased or increased endogenous HDAC4 levels respectively, but did not alter muscle mass or myofiber size. We subsequently manipulated miR-206 levels in muscles undergoing follistatin-induced hypertrophy or denervation-induced atrophy (models of muscle adaptation where endogenous miR-206 expression is altered). Vector-mediated manipulation of miR-206 activity in these models of cell growth and wasting did not alter gain or loss of muscle mass respectively. Our data demonstrate that although the miR-206/HDAC4 axis operates in skeletal muscle, the post-natal expression of miR-206 is not a key regulator of basal skeletal muscle mass or specific modes of muscle growth and wasting. These studies support a context-dependent role of miR-206 in regulating hypertrophy that may be dispensable for maintaining or modifying the adult skeletal muscle phenotype – an important consideration in relation to the development of therapeutics designed to manipulate microRNA activity in musculature.

## Introduction

Naturally-occurring, ~22 nucleotide long microRNAs (miRNAs, miRs) influence the translation and degradation of coding mRNA transcripts via sequence-specific interactions that promote RNA-interference [1]. Specific miRNAs have been identified as critical regulators of cellular processes that are essential for myogenic commitment and muscle formation during embryogenesis [2,3]. In the post natal-setting, a considerable body of work has identified roles for miRNAs in regulating cell size in cardiac musculature [4–7]. However, it has not yet been determined whether miRNAs perform a similar role in regulating the size of skeletal muscle fibers.

Of the microRNAs that are predominantly expressed in skeletal muscle, miR-206 has been defined as a key promoter of myogenic commitment, and of muscle regeneration following injury [8–10]. Augmenting the pro-myogenic effect of miR-206 has been considered as a therapeutic strategy to combat rhabdomyosarcoma [11], or ameliorate the pathology of neuromuscular disorders such as amyotrophic lateral sclerosis (ALS) [12] and muscular dystrophies [13–15]. In Texel sheep bearing a genomic mutation that creates an “illegitimate” miR-206 target site in the 3’ untranslated region of the myostatin gene, it has been demonstrated that miR-206 can also promote increased muscularity via myostatin repression during development and maturation [16]. These studies point to molecular mechanisms by which miR-206 could conceivably regulate the growth of skeletal muscle after development. However, it remains unclear to what extent the expression of miR-206 actually regulates cell size in post-natal skeletal muscle.

Histone deacetylases (HDACs) oppose the actions of histone acetyltransferases (HATs), by deacetylating nucleosomal histones, thereby promoting the condensation of chromatin and modifying the transcriptional of condensed genes [17]. Nuclear export, degradation, or pharmacological inhibition of HDACs can repress HDAC-mediated histone modifications. HDAC4 is a class II histone deacetylase that is highly expressed in striated muscle [18] and has been well characterized for its ability to bind and repress the myogenic transcription factor Mef-2 [19]. Class II HDACs have also been implicated in the regulation of cardiac and skeletal muscle growth [20,21]. Whilst we and others have shown that miR-206 targets HDAC4 to promote myogenic differentiation [12,22], it remains to be established whether miR-206 might also target HDAC4 to exert a positive influence upon cell size in post-mitotic skeletal muscle.

Our findings reported herein demonstrate that repression of miR-206 can promote hypertrophy associated with increased protein synthesis in cultured myotubes. Inhibition of HDAC4 activity *in vitro* prevented this mode of cell hypertrophy, indicating that the miR-206-HDAC4 axis plays a prominent role in the control of growth post mitotic cells of the myogenic lineage. In contrast, the administration of an rAAV6 vector encoding miR-206, or a miR-206-sponge construct designed to inhibit endogenous miR-206 activity did not affect basal muscle mass in adult mice, or affect myofiber size during episodes of experimentally-induced hypertrophy and atrophy, despite changes to endogenous levels of miR-206 in these states. Our data demonstrate that miR-206 is a context-dependent negative regulator of cell size in the myogenic lineage, but that the miR-206-HDAC4 axis appears to be dispensable for regulation of post-natal muscle mass *in vivo*.

## Experimental Procedures

### Ethics Statement

All experiments using animals were conducted in accordance with the relevant codes of practice for the care and use of animals for scientific purposes (National Institutes of Health, 1985, and the National Health & Medical Council of Australia, 2004). All experimental protocols were approved by the Alfred Medical Research and Education Precinct Animal Ethics Committee (AMREP AEC). All animal surgery was performed under inhalation of isoflurane in medical oxygen with post-operative analgesia, and all efforts were made to minimize animals’ discomfort.

### Antibodies and reagents

Antibodies against HDAC4 and α-tubulin were obtained from Cell Signaling, and an antibody against GAPDH was obtained from Santa Cruz. The anti-H3 acetylation antibody, sodium butyrate (SB) and valproic acid (VPA) were a kind gift from Dr A. El-Osta (Baker IDI Heart and Diabetes Institute, Australia). Oligonucleotides encoding inhibitors and mimics of miR-1, 206, 133 and a negative control were obtained from Dharmacon. Anti-miR-29 oligonucleotides and mimics including controls were obtained from Ambion. The negative control oligonucleotides (*C. Elegans* miRNA sequence not expressed in mice) were conjugated to a red fluorescent protein for visualization purposes. Laboratory chemicals were obtained from Sigma unless otherwise stated.

### Design and cloning of recombinant AAV vectors

AAV: miR-206 was designed by using the primary sequence of mouse miR-206 including 100bp upstream and downstream flanking region (synthesized by GenScript). For the AAV: miR-206 sponge, eight repeats of the previously validated 206 target site in the utrophin 3’ UTR [2] were arranged in series (Genscript). These fragments and the coding sequence for follistatin-288 (sourced from Open Biosystems) were individually cloned in into an AAV expression plasmid consisting of a CMV promoter/enhancer and SV40 poly-A region flanked by AAV2 terminal repeats (See Figure 3a) [23], using standard cloning techniques. Transfection of these plasmids with the pDGM6 packaging plasmid into HEK293 cells (a generous gift of Dr J.S. Chamberlain, University of Washington, Seattle) generated type-6 pseudotyped viral vectors that were harvested and purified as described previously [23]. Briefly, HEK293 cells were plated at a density of 3.2–3.8×10^6^ cells on a 10-cm culture dish, 8–16hr prior to transfection with 10 µg of a vector-genome-containing plasmid and 20 µg of the packaging/helper plasmid pDGM6, by means of the calcium phosphate precipitate method to generate pseudotype 6 vectors. Seventy-two hours after transfection, the media and cells were collected and homogenized through a microfluidizer (Microfluidics) prior to 0.22-µm clarification (Millipore). The vector was purified from the clarified lysate by affinity chromatography over a HiTrap heparin column (Amersham), and ultracentrifuged overnight prior to re-suspension in sterile physiological Ringer’s solution. The purified vector preparations were titered with a customized sequence-specific quantitative PCR-based reaction (Applied Biosystems Inc.) as described previously [24].

### Validation of AAV:miR and AAV:miR-sponge function

AAV: miR-206 and the AAV: miR-206 sponge were validated by testing their ability to regulate the HDAC4 3’ UTR *in vitro* using C2C12 myoblast cell cultures (ATCC) according to our methodology reported previously [22]. The entire HDAC4 3’ UTR was used to examine the effect of miR-206 by cloning the 3’ UTR into the pmiRGLO miRNA Target Expression Vector (Promega) [22]. Briefly, C2C12 myoblasts were seeded into 12-well plates and transfected with the HDAC4 3’ UTR (full length), a *LacZ* plasmid and either AAV: miR-206 or the AAV: miR-206 sponge using Lipofectamine 2000. Cells were incubated for 48 hr prior to assaying luciferase activity and β-galactosidase activity using commercially available reaction substrates (Promega).

### Western blotting

TA muscles were homogenized in RIPA-based lysis buffer (Millipore) with CompleteTM EDTA-free protease and phosphatase inhibitor cocktails (Roche). C2C12 myotubes were lysed using Triton-X based lysis buffer (30mM Hepes, 150mM NaCl, 1% Triton-X-100, 2mM MgCl_2_), with CompleteTM EDTA-free protease and phosphatase inhibitor cocktail (Roche). Lysis was followed by centrifugation at 13000 × g for 10 min at 4^°^C and samples were denatured for 5 min at 95^°^C. Protein concentration was determined using a Pierce micro protein assay kit (Thermo-Scientific). Protein fractions were subsequently separated by SDS-PAGE using pre-cast 4-12% Bis-Tris gels (Life Technologies), blotted onto nitrocellulose membranes (BioRad) and incubated with the appropriate antibody overnight and detected as described previously [22]. Quantification of labeled Western blots was performed using ImageJ pixel analysis (NIH Image software), and data are normalized to a control value of 1. Densitometric analyses of Western blots are presented as band density normalized to the loading control, and are representative of at least three independent experiments.

### Histochemical staining

Harvested muscles were placed in OCT cryoprotectant and frozen in liquid nitrogen-cooled isopentane. The frozen samples were subsequently cryosectioned at 10 µm thickness and stained with hematoxylin and eosin to examine morphology as described previously [25]. Sections were fixed in methanol, rinsed in distilled water, immersed in hematoxylin solution (Amber Scientific, Australia) for 3 min, dip-rinsed in distilled water and tap water, incubated in Scott’s tap water (Amber Scientific, Australia) for 1 min, followed by running tap water for 2 min, then immersed in Eosin solution (Amber Scientific, Australia) for 2 min, and subsequently transferred through increasing strengths of ethanol before immersion in xylene, and coverslipping with DEPEX (BDH) mountant. The minimum Feret’s diameter of myofibers, and the mean width of myotubes was determined using ImageJ software. The width of at least 200 myotubes was counted in total per treatment from at least 4 different wells and three fields per well. For quantification of myofiber diameter from skeletal muscle, the minimum Feret’s diameter was calculated by counting at least 500 myofibers from 3 fields and this was repeated for three separate muscles per treatment.

### Fusion Index

Differentiated myotubes treated with, or without, hp-miR-206 were fixed with ethanol at room temperature and stained with Hematoxylin. Nuclei were counted in twelve randomly chosen microscope fields (4 wells, 3 fields per treatment). One microscope field usually contained between 300 and 400 nuclei. The fusion index was calculated as the number of nuclei in myotubes divided by the total number of nuclei per well as described previously [26,27].

### miRNA transfections and reagents

C2C12 cells were cultured in Dulbecco’s Modified Eagle’s Medium (DMEM, Life Technologies) containing 10% fetal bovine serum (FBS; HyClone), 2 mM glutamine, 100 U/ml penicillin and 100 µg/ml streptomycin at 37^°^C, whilst gassed with 10% CO_2_-supplemented air. To differentiate cells into myotubes, media was changed to DMEM substituted with 2% horse serum in place of FBS. Cells were cultured under these conditions for 4-5 days and media was changed every second day. Hairpin and mimic miRNA sequences were transfected in 12-well plates using Metafectene Pro (Biontex) in media devoid of antibiotics. Media was changed 4-6 hr after transfection and myotubes were tracked for up to 96 hr post-transfection. Initial experiments were carried out using 200nM of mimic or inhibitor as recommended by Dharmacon and further experiments used doses ranging from 40 to 200 nM. For experiments using VPA (2.5mM and 5mM) and SB (2.5 mM and 5 mM) to inhibit HDAC activity, myotubes were initially transfected with the miRNAs, and after 4 hr, media was removed and myotubes were treated with the appropriate inhibitor for 48 hr.

### Quantitative RT-PCR

Total RNA was collected from C2C12 myotubes or snap frozen tissue, using Trizol (Life Technologies). 500-1000 ng of RNA was reverse transcribed using the High Capacity RNA-to-cDNA kit (Applied Biosystems). cDNA was subsequently analyzed by quantitative RT-PCR using SYBR Green or TaqMan^TM^ master mixes (both from Applied Biosystems). MyoD primers for use with SYBR Green (F: GTAGCGGAGACTCGGAATTG; R: GAAGTTCTGAGGTGGCAAGC) were obtained from Life Technologies. *18S* was used to standardize for cDNA concentrations (probe and primers from Applied Biosystems). For miRNA analysis, RNA was isolated according to the standard Invitrogen protocol and quantitated using the NanoDrop^TM^ fluorospectrometer (Thermo Scientific). Assay kits for each miRNA were purchased from Applied Biosystems and amplified according to the manufacturer’s instructions. Data were analyzed using the ΔΔCT method of analysis and are representative of at least three independent experiments.

### Ex vivo protein synthesis

Protein synthesis was measured as described previously [28]. C2C12 myotubes were treated with miR-206 mimic or inhibitor in differentiation media for 24 hours. For the final hour pre-harvest, media was supplemented with 1 µCi of ^3^H-tyrosine (GE Health Care, UK) and 2 mM L-tyrosine (pH 7.4). At harvest, the myotubes were washed twice in ice-cold PBS and lyzed in 1.5 ml of 10% (v/v) Trichloroacetic acid. The lysates were transferred into Eppendorf tubes and allowed to precipitate for 1 h on ice, centrifuged for 10 min at 20,000 × g then dissolved overnight at room temperature in 500 µl of 0.1 M NaOH and 1% Triton X-100. To estimate tyrosine incorporation, 400 µl of lysate was subsequently added to 4 ml of scintillation fluid and measured using a scintillation counter (PerkinElmer). Protein concentration was determined using a BCA protein assay (Thermo Scientific) and used to normalize the scintillation counts. Protein synthesis is presented as scintillation counts per minute/mg of protein.

### Experimental animals and surgical procedure

All experiments were conducted in accordance with the relevant codes of practice for the care and use of animals for scientific purposes (National Institutes of Health, 1985, and the National Health & Medical Council of Australia, 2004). For local vector delivery, 8 week old C57Bl/6 mice were deeply anesthetized with isoflurane, and 1×10^9^ or 1×10^10^ vector genomes of AAV-miR-206, AAV-miR-206 sponge or AAV-follistatin-288 (~5×10^9^ vg) were injected in 30 µl of Hank’s buffered saline solution (HBSS) directly into the anterior compartment of the hindlimb which is occupied by the tibialis anterior (TA) muscle. Control-injections of the contralateral limb used a vector lacking a functional gene (referred to as Control). For denervation studies, mice were deeply anesthetized, and 1-2mm of the peroneal nerve was surgically excised to denervate the TA muscle. For tissue harvest, mice were humanely killed via a cervical dislocation, and the muscles rapidly excised before subsequent processing. For analyses of mouse tissues following treatment, at least 4 mice were used per cohort.

### Statistical Analysis

The Students T-test, or One/Two Way ANOVA (with the Student-Newman-Keuls post-hoc) were used where appropriate. Data are reported as the mean ± S.E.M.

## Results

### Inhibition of miR-206 promotes myotube hypertrophy in vitro

**Figure 1:**
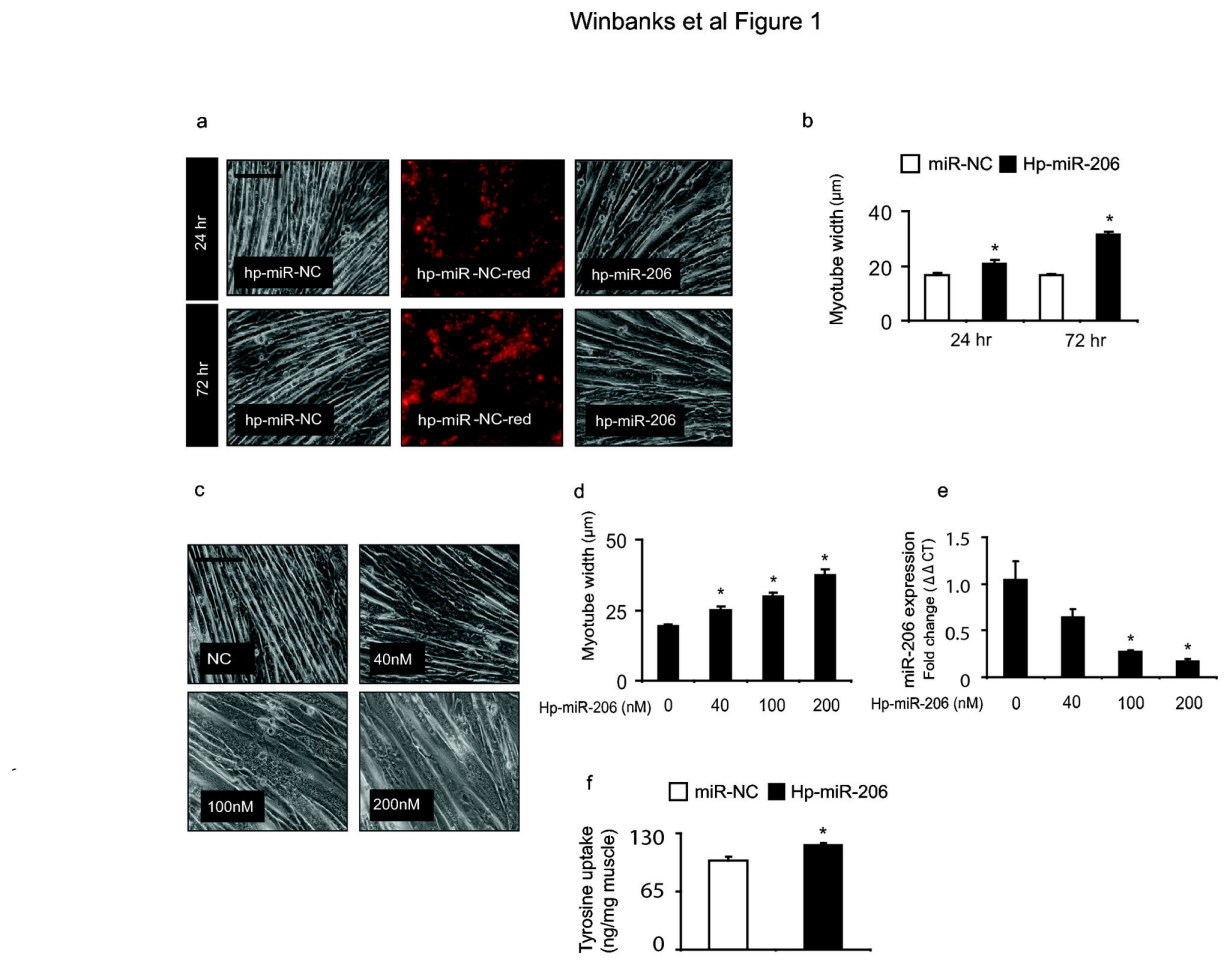
Hairpin mediated inhibition of miR-206 results in robust skeletal muscle hypertrophy in vitro (a) Inhibition of miR-206 in fully differentiated C2C12 myotubes increased cell diameter after 24 and 72 hr of treatment. The fluorescent conjugated control demonstrates the high transfection efficiency. n=4 per group representative of three independent experiments. Scale, 100µm (b) Myotubes were quantified by measuring myotube diameter. Inhibition of miR-206 increased myotube diameter ~25% after 24hr (*, p<0.001 vs. control) and ~90% after 72hr (*, p<0.001 vs. control). n=3 per group representative of three independent experiments (c) Myotube hypertrophy correlated with the dose of miR-206 inhibitor administered. Scale, 100µm (d) miR-206 inhibition increased myotube diameter ~30%, 55% and 95% in response to 40 nM, 100 nM or 200 nM of miR-206 inhibitor (*, all p<0.001 vs. control) n=3 per group representative of three independent experiments. (e) Expression of the hairpin inhibitors resulted in concordant reductions in the transcriptional activity of miR-206 that were significant at 100 nM and 200 nM (*, all p<0.02 vs. control) n=3 per group representative of three independent experiments (f) *Ex vivo* tyrosine uptake was measured in myotubes treated with miR-206 inhibitor and as compared to cells treated with the negative control (miR-NC), protein synthesis was increased by 20% (*, p=0.003 vs. control) (n=6 per group repeated three times).

To determine whether miR-206 levels influence cell size in differentiated musculature, we applied exogenous miR-206 mimics and inhibitors to differentiated myotube cultures. Whilst over-expression of miR-206 did not affect myotube size (Figure S1a), the hairpin mediated inhibition of miR-206 caused a time-dependent increase in myotube width (Figure 1a), which was significantly increased from 24 hr treatment (Figure 1b). Furthermore, the hypertrophic response to miR-206 inhibition increased in accordance with inhibitor dosage, such that myotube width was increased by 30%, ~55% and ~95% in response to 40nM, 100 nM and 200 nM doses of miR-206 inhibitor (Figure 1c-d). Increases in myotube dimensions correlated with significant reductions in miR-206 transcript levels at 100 nM and 200 nM (Figure 1e). As myotube width was increased in response to inhibition of endogenous miR-206, we examined whether treated myotubes had increased rates of protein synthesis. As shown in Figure 1f, tyrosine uptake into myotubes was significantly increased 24hr after inhibition of miR-206, suggesting the recruitment of protein synthesis pathways contributed to the promotion of growth following miR-206 inhibition. To elucidate whether the observed myotube hypertrophy was dependent on increases to myoblast fusion, we then measured the fusion index in cultures treated with 200nM hp-miR-206. We did not identify changes to the fusion index after 48 hr when this treatment was compared to control treated myotubes (Figure S1b). Taken together these data demonstrate that miR-206 can specifically and robustly regulate growth of post mitotic cells of the skeletal muscle lineage, at least in part by the regulation of protein synthesis networks. These effects are specific to the inhibition of miR-206, as the over-expression or inhibition of other miRNAs that are enriched in muscle, including miR-133a and the miR-29 family did not have any effect on morphology in similarly differentiated myotubes (Figure S2a-b).

### Myotube hypertrophy induced by the inhibition of miR-206 is regulated by HDAC4

**Figure 2:**
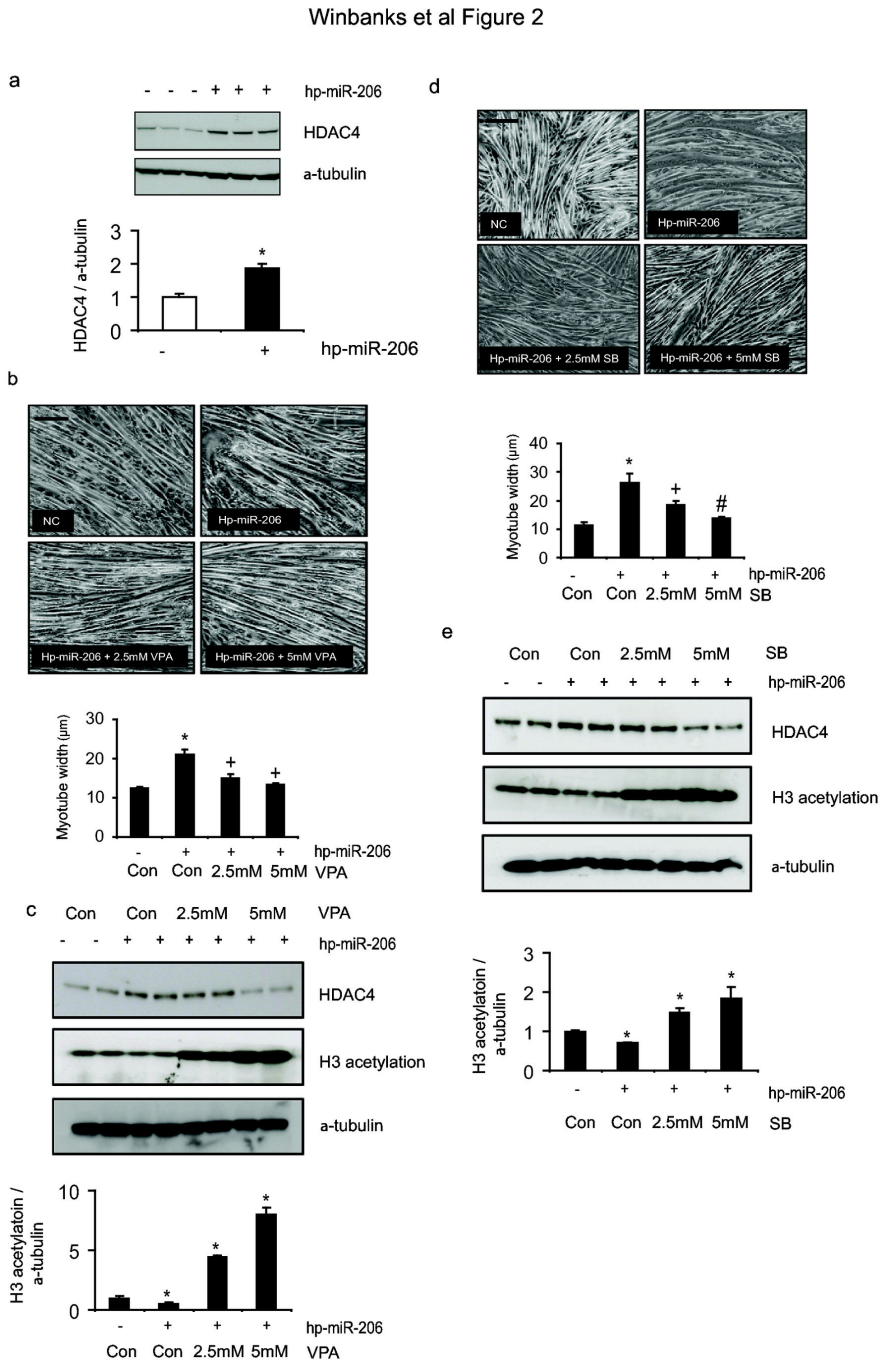
Hypertrophy induced by reduction of miR-206 is regulated by HDAC4. (a) HDAC4 protein levels were assessed 24hr after transfection of miR-206 inhibitor (hp-miR-206) into differentiated myotubes (*, p=0.0006 vs. control) n=3 per group repeated three times (b) Fully differentiated myotubes were transfected with the NC or hp-miR-206 and after 4 hr treated with vehicle or increasing doses of the HDAC4 inhibitor VPA for 48hr. n=3 per group repeated three times. Myofiber diameter was calculated by measuring the width of at least 200 myotubes per treatment. Scale, 100µm (*, p<0.001 vs. control, ^+^, p<0.001 vs. hp-miR-206). (c) H3 acetylation (*, p<0.05 vs. control) was assessed as a marker of HDAC activity by Western blot (*, p<0.05 vs. control). (d) To confirm the effect of HDAC inhibition by valproic acid (VPA), myotubes were subsequently treated with increasing doses of sodium butyrate (SB) – another HDAC inhibitor - and after 48hr the width of myotubes was assessed (*, p<0.001 vs. control, ^+^, p<0.001 vs. hp-miR-206, ^#^, p=0.002 vs. hp-miR-206). Scale, 100µm (e) H3 acetylation levels were assessed via Western blot (*, p<0.05 vs. control).

The 3’ UTR of HDAC4 is a key target of miR-206 in the control of myogenic differentiation [22] and satellite cell recruitment [13]. We therefore sought to determine whether the miR-206-HDAC4 pathway is conserved in post-mitotic myotubes and whether this axis regulates the cell hypertrophy induced by inhibition of miR-206. As shown in Figure 2a, hairpin mediated inhibition of miR-206 in differentiated myotubes led to increases in HDAC4 protein levels after 24hr treatment. We have shown previously that this mechanism involves targeting of the HDAC4 3’ UTR by miR-206 [22]. Reductions in miR-206 would therefore relieve suppression of the HDAC4 3’ UTR by miR-206, subsequently increasing HDAC4 protein levels. We therefore sought to determine whether inhibition of HDAC4 could prevent the growth-promoting effects associated with miR-206 inhibition. As shown in Figure 2b, treatment of myotubes with valproic acid (VPA), a potent class II HDAC inhibitor [29] prevented hypertrophy, and increases in myotube diameter, induced by miR-206 inhibition. We confirmed the ability of VPA to inhibit the enzyme activity of histone deacetylases by measuring H3 acetylation, which was significantly increased in response to both doses of VPA (Figure 2c). We also found that inhibition of miR-206 inhibited H3 acetylation (Figure 2c). These results were further confirmed with sodium butyrate (SB), another class II HDAC inhibitor [30]. As shown in Figure 2d, treatment with increasing doses of SB prevented the increases in myotube diameter induced by miR-206 inhibition. The efficacy of HDAC4 inhibition by SB, and of the miR-206 inhibitor, was confirmed by measuring changes in histone H3 acetylation (Figure 2e). These data demonstrate that stimulation of myotube hypertrophy subsequent to miR-206 inhibition involves de-repression of histone deacetylase activity.

### AAV vectors encoding miR-206 or a miR-206 sponge effectively regulate miR-206 levels in vivo

**Figure 3:**
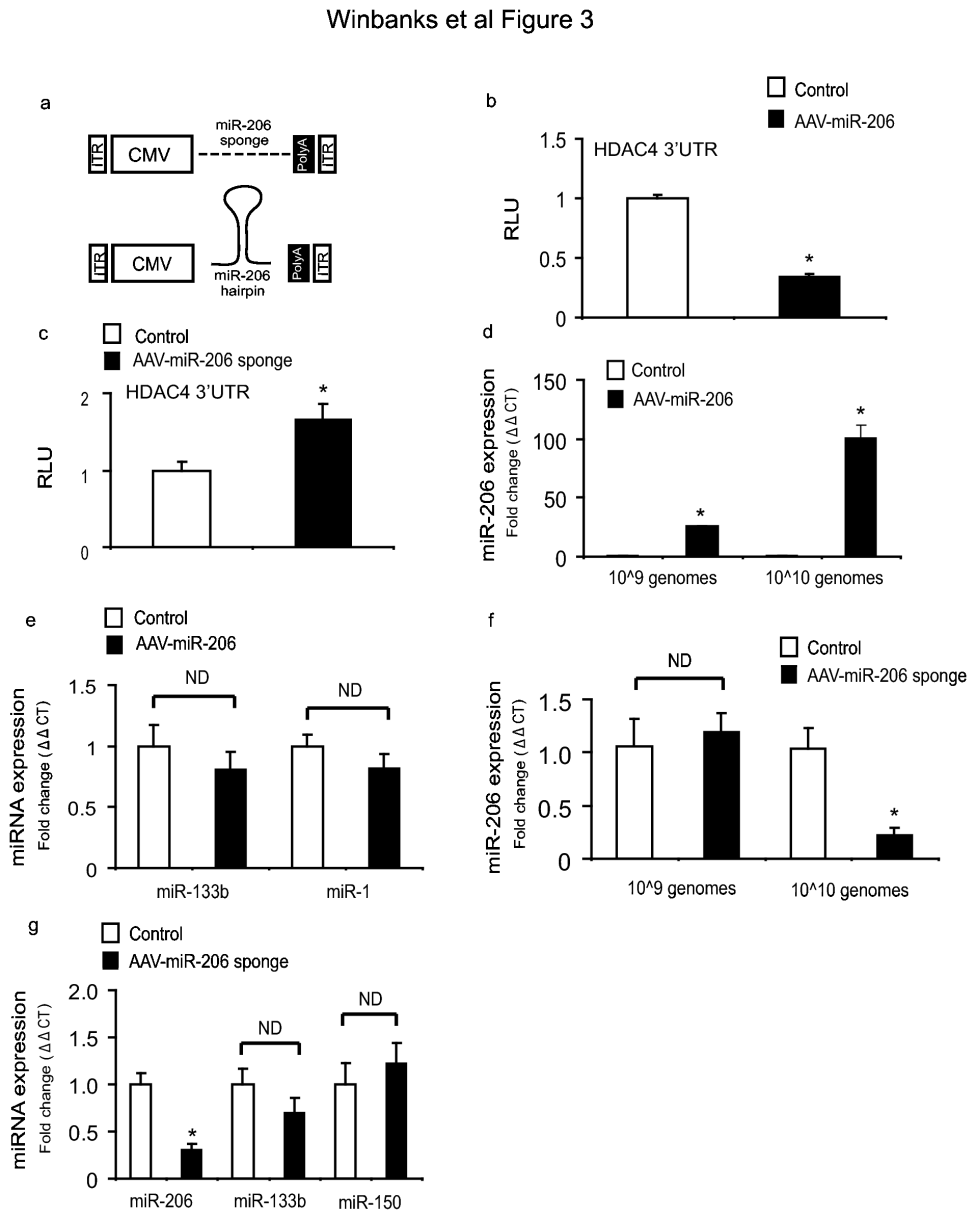
Design and validation of AAV:miRNA-206 and AAV:miR206-sponge vectors. (a) The design of AAV vectors encoding either miR-206 or a miR-206-sponge (see Methods for details). (b–c) To confirm function, miR-206 and miR-206-sponge constructs were tested for ability to regulate the HDAC4 3’ UTR *in vitro* using C2C12 cells. After 48 hr, cells were lysed and luciferase activity was measured and normalized to β-gal activity. Whilst AAV: miR-206 inhibited HDAC4 3’ UTR activity by 65% (*, p<0.01 vs. control), the AAV: miR-206-sponge increased the activity of the HDAC4 3’ UTR by 60% (*, p<0.05 vs. control, n=3 per group repeated three times). (d) Administration of 1×10^9^ and 1×10^10^ vector genomes of AAV: miR-206 *in vivo* increased miR-206 transcription approximately 20 fold (*, p=0.002 vs. control) and 100 fold (*, p<0.001 vs. control) respectively, n=3 per group repeated three times. (e) AAV: miR-206 was injected into the TA muscles of mice and after 28 days, miR-133b and miR-1 levels were measured by RT-PCR (p=ND, n=8). (f) AAV: miR-206-sponge vector (1×10^10^ vector genomes administered) reduced miR-206 transcripts, as determined by RT-PCR (*, p=0.03 vs. control, n= 4 per group). (g) Whilst the AAV: miR-206-sponge vector (1×10^10^ vector genomes administered) reduced miR-206 transcripts (*, p=0.04 vs. control), it did not affect expression of miR-133b or miR-150 (N=ND, n=3 per group).

Having identified that inhibition of miR-206 induces myotube hypertrophy *in vitro*, we next sought to test whether over-expression or inhibition of miR-206 played a role in regulating skeletal muscle growth *in vivo*. We developed recombinant AAV vectors carrying expression cassettes encoding either the primary miR-206 sequence, or eight repeats of the previously validated miR-206 target site found in the 3’ UTR of utrophin (Figure 3a and Methods). We tested the function of our vectors *in vitro* by assessing their ability to regulate the HDAC4 3’ UTR. Administration of AAV: miR-206 inhibited HDAC4 3’ UTR luciferase activity (Figure 3b), whereas AAV: miR-206-sponge quenched endogenous miR-206 levels and resulted in increased HDAC4 3’ UTR luciferase activity (Figure 3c). Having confirmed vector functionality *in vitro*, we administered vectors to the leg muscles of mice and found that administration of AAV: miR-206 *in vivo* at 1×10^9^ and 1×10^10^ vector genomes significantly increased miR-206 expression by 28 days post injection (Figure 3d). Importantly, we found that miR-206 over-expression via administration of AAV: miR-206 did not effect the expression of miR-133b, which forms a bicistronic pair with miR-206 [11], or miR-1 which shares high sequence homology with miR-206 (Figure 3e). Administration of the AAV: miR-206-sponge vectors to the muscles of mice did not affect miR-206 transcripts at 1×10^9^ vector genomes, but suppressed miR-206 transcriptional activity by approximately 80% at a dose of 1×10^10^ vector genomes (Figure 3f). In contrast, the AAV: miR-206 sponge did not effect the expression of miR-133b or miR-150 (Figure 3g). These results demonstrate that administration of AAV vectors encoding miR-206 or a miR-206 sponge are valid and effective methods to manipulate miRNA-206 levels *in vivo*.

### Over-expression or inhibition of miR-206 in vivo does not affect post-natal skeletal muscle growth

**Figure 4:**
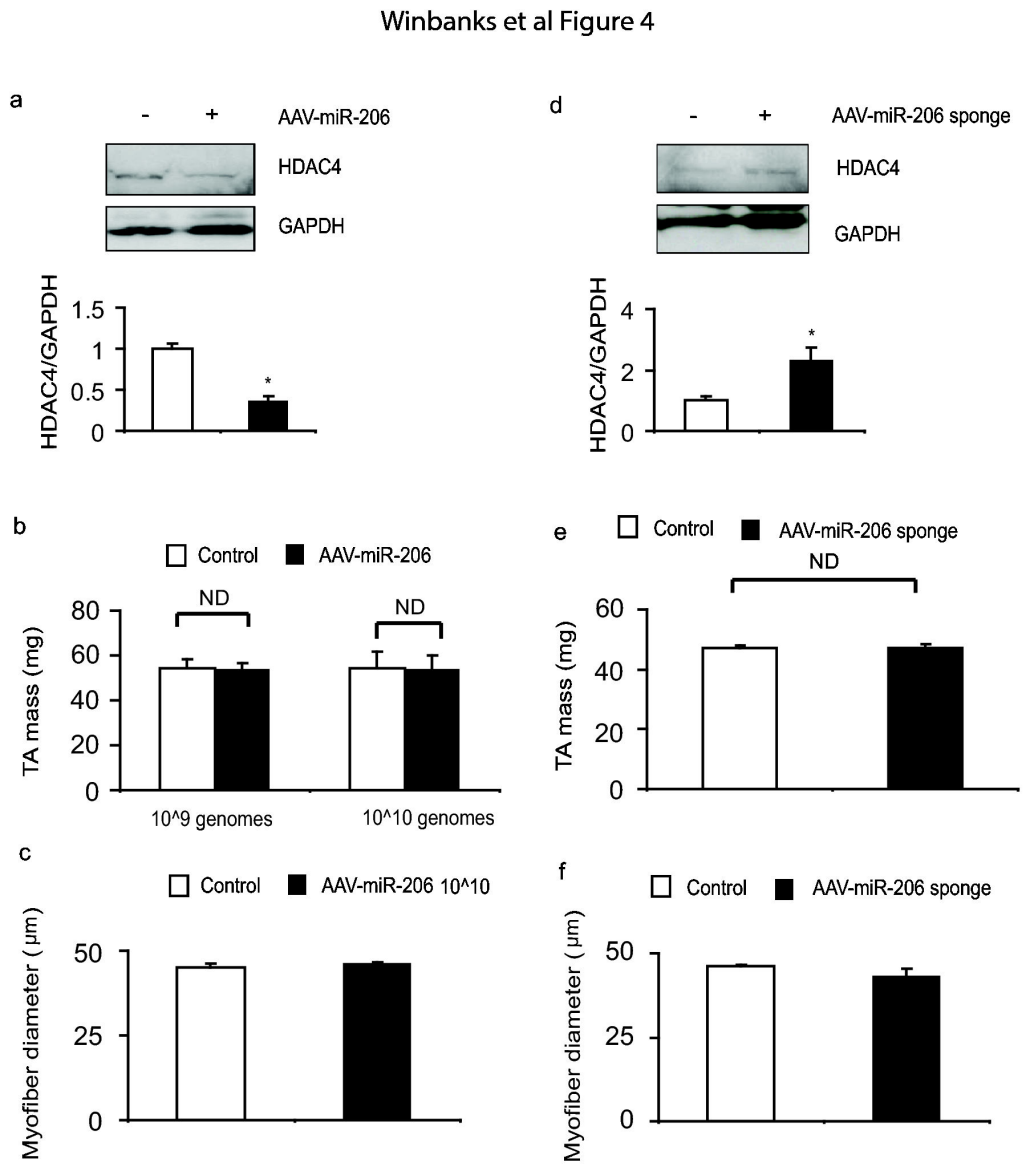
The administration of AAV:miR-206 or AAV:miR-206-sponge vectors does not affect post-natal skeletal muscle mass (a) HDAC4 protein levels were assessed 28 days after injection of mouse limb muscles with AAV: miR-206 (*, p<0.01 vs. control, n=3-5 per treatment). (b–c) TA muscle mass was assessed 28 days after injection of 1×10^9^ and 1×10^10^ vg of AAV: miR-206. No differences were observed in muscle mass or myofiber diameter between treated and control muscles, Myofiber diameter was assessed by measuring the minimum Feret’s diameter. n=4 per treatment. (d) HDAC4 protein levels were examined by Western blot (*, p=0.048 vs. control) in muscles examined 28 days after injection with injected with AAV: miR-206-sponge vector, n=4 per treatment. (e–f) TA muscle mass, and myofiber diameter, were not affected by administration of AAV: miR-206-sponge 28 days. n=4 per treatment.

Having successfully designed AAV vectors that can effectively manipulate miR-206 levels *in vivo*, we next tested whether vector-mediated manipulation of miR-206 levels could regulate skeletal muscle mass *in vivo*. In spite of the ability of AAV: miR-206 to reduce HDAC4 protein levels in skeletal muscle (Figure 4a), we found no effect of AAV: miR-206 administration upon TA muscle mass or myofiber diameter as compared to muscles treated with the control vector (Figure 4b-c). Similarly, in spite of the administration of AAV: miR-206-sponge vector increasing levels of HDAC4 *in vivo* we did not observe an effect of treatment upon basal muscle mass or myofiber diameter (Figure 4d–f). These data demonstrate that although the miR-206-HDAC4 axis is conserved *in vivo* in post natal mammalian skeletal muscle, miR-206 expression appears to be dispensable in the regulation of basal skeletal muscle mass.

### Endogenous expression of miR-206 changes inversely in muscle hypertrophy and atrophy but exogenous manipulation of miR-206 does not affect muscle adaptation

**Figure 5:**
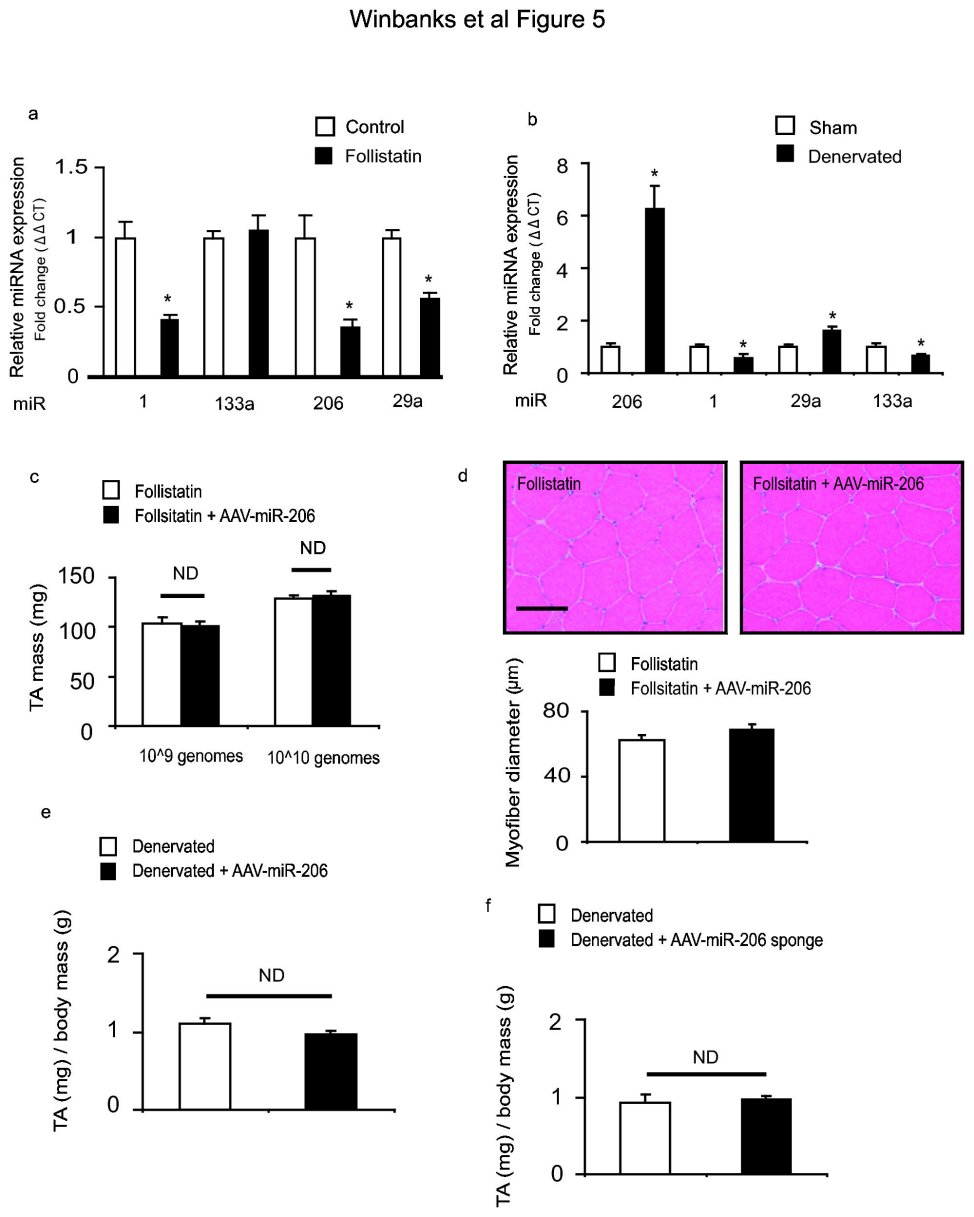
Modulation of miR-206 activity does not regulate skeletal muscle hypertrophy or atrophy (a) RNA from follistatin expressing muscles undergoing hypertrophy and control muscles was extracted and analyzed via RT-PCR. By 14 days, expression of miRNA-1 (*, p<0.01 vs. control), miR-206 (*, p<0.01 vs. control), and miR-29a (*, p<0.01 vs. control) were decreased, n=6 per treatment. (b) In association with muscle atrophy occurring 7 days after motor nerve resection, miR-206 (*, p<0.01 vs. control) and miR-29a (*, p=0.03 vs. control) expression was increased, whilst miR-133a (*, p=0.02 vs. control) and miR-1 expression was suppressed (*, p=0.02 vs. control). n=6 per treatment (c) Co-administration of AAV: Follistatin-288 and AAV: miR-206 for 28 days did not effect hypertrophy induced by follistatin-288. n=4-7 per treatment (d) Muscle cryosections stained with hematoxylin and eosin demonstrate consistent muscle fiber morphology and no changes in muscle fiber diameter n=8 per treatment. Scale, 100µm (e–f) The TA muscles of mice examined 14 days after nerve resection and administration of either AAV: miR-206 or AAV: miR-206 did not exhibit differences in muscle mass, or myofiber diameter, compared with denervated muscles receiving a control vector. Muscle mass was normalized over initial body weights, n=4 per treatment.

Having determined that modulation of miR-206 expression does not influence basal muscle mass *in vivo*, we sought to determine whether changes in miR-206 expression associated with muscle hypertrophy and atrophy are necessary for muscle adaptation to occur. Previous work has demonstrated the ability of TGF-β signaling to regulate miR-206 expression [22]. We therefore examined changes to miR-206 in response to an AAV vector encoding a modulator of TGF-β signaling – follistatin-288. Follistatin is a robust inducer of skeletal muscle hypertrophy when over-expressed *in vivo*, causing an approximate doubling of muscle mass in mice treated for 28 days [31]. In muscles examined 14 days after injection of a follistatin-expressing AAV vector, we observed reduced expression of miR-206, miR-1 and miR-29a (Figure 5a). Conversely in TA muscles undergoing atrophy 7 days following surgical resection of the peroneal nerve, miR-206 was robustly increased. Interestingly, miR-206 and miR-29a were the only miRNAs to show inverse changes in response to inducers of growth and atrophy respectively (Figure 5b). Furthermore, we found that MyoD, a known regulator of miR-206 expression [2], was inhibited in response to follistatin (Figure S3), but increased in response to denervation (Figure S3), suggesting that MyoD may regulate miR-206 expression in these models of muscle growth and atrophy. We subsequently tested whether increased miR-206 levels can inhibit muscle growth in vivo by co-administering AAV: follistatin with AAV: miR-206. As compared to the effect of follistatin alone, over-expression of miR-206 by either 20 fold (1×10^9^ vg dose) or 100 fold (1×10^10^ vg dose) did not prevent follistatin-induced hypertrophy (Figure 5c-d). Vector-mediated over-expression or inhibition of miR-206 in denervated muscles did not exacerbate or rescue the phenotype respectively (Figure 5e-f). These data demonstrate that although miR-206 expression is altered during skeletal muscle hypertrophy and atrophy, direct modulation of miR-206 activity does not appear to play an essential role in facilitating skeletal muscle growth or wasting.

## Discussion

Specific microRNAs are increasingly being characterized as pivotal regulators of myogenic commitment, muscle formation, and skeletal muscle adaptation [5,30,31]. Since miR-206 was identified as an important promoter of myogenic differentiation [9], a number of studies have profiled the response of miR-206 in models of skeletal muscle atrophy or hypertrophy in an attempt to determine its role in regulating post-natal adaptation of skeletal muscle. Expression of miR-206 increases in muscles of *mdx* mice (a model of dystrophinopathy) [15,32], in ALS and sarcopenia [12,33], following hypogravity exposure during space flight [34], and during hibernation [32], thereby supporting a role for miR-206 in the regulation of muscle mass. However, because miR-206 expression was reduced with TWEAK-induced muscle wasting [33], and increased in muscles undergoing hypertrophy due to ablation of myostatin [34], the role that miR-206 plays in these models is less clear. In Texel sheep, miR-206 appears to promote the hypermuscular phenotype by repressing myostatin, as a consequence of a polymorphism that creates a miR-206 target site in the 3’ UTR of the myostatin gene [16]. However, the role of miR-206 expression upon the regulation skeletal muscle growth and post-natal adaptation elsewhere has not yet been elucidated. The findings reported herein demonstrate that inhibition of miR-206 *in vitro* promotes hypertrophy of cultured myotubes via de-repression of HDAC4, a previously confirmed target of miR-206 [12,22]. Interestingly, we did not observe myotube atrophy subsequent to over-expression of miR-206 *in vitro*. This result is likely due to the expression of miR-206 already being very high as a function of myogenic differentiation and myotube formation (data not shown), and therefore the growth-repressing effects of miR-206 already being maximal at endogenous expression levels [15,38]. In contrast to miR-206, we found that manipulation of miR-133a and the miR-29 family in highly differentiated myotubes had no effect on myotube hypertrophy, in spite of their role in the regulation of cardiomyocyte growth [4]. These findings demonstrate that miR-based effects on cell growth are not always conserved between cardiac and skeletal musculature.

HDACs are commonly associated with the inhibition of gene expression, by antagonizing histone acetyltransferase (HAT) activity [19]. Class II HDACs have been implicated in both the repression and the promotion of growth in cardiac musculature [20,35,36]. Our studies demonstrate that the hypertrophy of cultured myotubes induced by inhibition of miR-206 was mediated through de-repression of HDAC4, as this hypertrophic effect could be inhibited via Class II HDAC inhibition. miR-206 has also been shown to target connexin 43, which promotes fusion of myoblasts [8]. A reduction of miR-206 in myotubes could therefore promote myotube hypertrophy via increased fusion of adjacent myoblasts to expand the cytoplasmic volume of myotubes. However, as myotube hypertrophy induced by miR-206 inhibition was prevented by the administration of Class II HDAC inhibitors, these data support a miR-206 – HDAC4 interaction as the dominant mechanism underlying the observed regulation of myotube size.

Consistent with earlier studies that developed the miR-sponge approach [37] our findings demonstrate that the administration of a miR-206 sponge construct comprising multiple miR-206 target sites is an effective means of inhibiting endogenous miR-206 activity. Moreover, we have shown that this technology can be employed to manipulate miR activity in mammalian muscles when delivered in a recombinant AAV vector. However, although we were successful in developing AAV: miR and AAV: miR-sponge vectors that can increase or decrease miR-206 activity *in vivo* respectively, it is remarkable to consider that these interventions did not affect the basal regulation of mass in the skeletal muscles of young-adult mice, despite vector-mediated miR manipulation altering HDAC4 protein expression. These data suggest that the miR-206/HDAC4 axis is dispensable in regulation of post-natal skeletal muscle mass. From these findings we hypothesized that miR-206 may play a more significant role in regulating skeletal muscle adaptation. Others have reported that in cardiac musculature, particular microRNAs may operate as regulators of adaptation more so than regulators of the basal state [38]. We observed decreased miR-206 expression associated with follistatin-mediated muscle hypertrophy, and significantly increased miR-206 expression during denervation induced atrophy (as per previous studies [12]). However, neither vector-mediated miR-206 over-expression, nor miR-206 inhibition altered the response of muscles to follistatin, or to nerve resection. These data demonstrate that the miR-206/HDAC4 axis does not appear to play an essential role in regulating these specific modes of skeletal muscle hypertrophy and atrophy.

In conclusion, this study has identified a context-dependent mechanism by which miR-206 potently limits the size of cells in the myogenic lineage by inhibiting the activity of HDAC4. That we observed changes in endogenous miR-206 expression associated with the hypertrophy and atrophy of murine muscles, but that vector-mediated manipulation of the miR-206/HDAC4 axis *in vivo* did not alter post-natal muscle mass or adaptive responses demonstrates that changes in miR-206 expression alone are not sufficient to regulate skeletal muscle mass after development. We hypothesize that the discrepancy between the effects observed *in vitro* and *in vivo* is attributed in part to the added complexity of stimuli that regulate the phenotype of skeletal muscle *in vivo*. However, this context-dependent role of miR-206 sits in contrast with previous findings that have demonstrated other miRs can regulate the post-natal adaptation of cardiac musculature [38], or skeletal muscle [39], and suggests that the dominant role of miR-206 in muscle is associated with the process of muscle development [40]. Such a notion is supported in part by the role attributed to miR-206 in establishing the hypermuscular attributes of Texel sheep during development [16]. Our results do not exclude the possibility that miR-206 can still contribute to the on-going regulation of the skeletal muscle phenotype by acting in concert with other microRNAs expressed in muscle. microRNAs are differentially expressed during skeletal muscle adaptation, so it is plausible that cohorts of specific microRNAs may need to be jointly manipulated to influence the expression of genes that determine the phenotype of skeletal muscle. As interventions that can manipulate endogenous microRNA activity are being investigated as prospective therapeutics for diseases affecting many tissues including striated musculature, it is important that the results presented here be considered as part of ongoing work to comprehensively define the role of microRNAs in the post-developmental regulation of cell homeostasis and adaptation.

## Supporting Information

Figure S1 **Increased miR-206 expression does not affect myotube morphology.** (a) Differentiated C2C12 myotubes were transfected with mimics for miR-206. A fluorophore-conjugated miRNA not expressed in mice was used as a negative control (miR-NC). 4-6 hr later, media was replaced and cells were tracked for morphology 48 hr post-transfection, n=3 per treatment, repeated three times. Scale, 100µm. (b) Differentiated C2C12 myotubes were transfected with hp-miR-206 or negative control (miR-NC) as outlined in (a). Myofibers were stained with hematoxylin. Fusion index was determined by counting hematoxylin stained nuclei in myotubes and dividing that number by the total number of nuclei per field counted. Data are presented as a ratio. n=ND.
(EPS)

Figure S2 **Expression or inhibition of miR-133a or the miR-29 family does not affect myotube hypertrophy in vitro.** (a) Differentiated C2C12 myotubes were transfected with mimics for miR-133a or hairpin oligonucleotides against 133a. A fluorophore-conjugated miRNA not expressed in mice was used as a negative control (miR-NC). 4-6 hr later, media was replaced and cells were tracked for morphology 48 hr post-transfection, n=3 per treatment, repeated three times. Scale, 100µm (b) As outlined in (a) fully differentiated myotubes were transfected with oligonucleotides that act to inhibit miR-29abc or activate miR-29abc, along with the appropriate negative control (miR-NC), n=3 per treatment, repeated three times. Scale, 100µm.
(EPS)

Figure S3 **MyoD expression is altered in association with follistatin-mediated hypertrophy and denervation-induced wasting of mouse limb muscles.** Injection of muscles with AAV: Follistatin-288 subsequently reduced expression of MyoD (*, p<0.05 vs. control, n=6 per treatment), whereas denervation of muscles results in increased MyoD expression (*, p<0.05 vs. control, n=6 per treatment).
(EPS)
